# Supplementary material for: Epigenetic weapons in plant-herbivore interactions: Sulforaphane disrupts histone deacetylases, gene expression, and larval development in Spodoptera exigua while the specialist feeder Trichoplusia ni is largely resistant to these effects
Source: PLoS One. 2023 Oct 19;18(10):e0293075. doi: 10.1371/journal.pone.0293075 (PMC10586618; doi:10.1371/journal.pone.0293075)
Supplement: S1 Table — (DOCX) [file pone.0293075.s001.docx]

**Supplementary Information Appendix for:**

**Epigenetic weapons in plant-herbivore interactions: Sulforaphane disrupts lepidopteran histone deacetylases, gene expression, and larval development**

Dana J. Somers, David B. Kushner, Alexandria R. McKinnis, Dzejlana Mehmedovic, Rachel S. Flame, and Thomas M. Arnold*

Department of Biology, Program in Biochemistry and Molecular Biology, Dickinson College, Carlisle, PA USA 17013

*corresponding author: [arnoldt@dickinson.edu](mailto:arnoldt@dickinson.edu)

**S1 Table. Expression of HDAC genes in *S. exigua* and *T. ni.***

| Class | Gene | SFN ^a^ | | TSA ^a^ | |
| --- | --- | --- | --- | --- | --- |
|  |  | ***S. exigua*** | ***T. ni*** | ***S. exigua*** | ***T. ni*** |
| Class 1 | HDAC2 | 0.53 | 0.40 | 0.38 | 0.13 |
|  | HDAC3 | 0.38 | 0.87 | 0.27 | 0.39 |
| Class 2 | HDAC7 | 0.12 | 0.24 | -0.09 | 0.25 |
|  | HDAC6 | 1.04 | -0.16 | 0.74 | -0.42 |
| Class 4 | HDAC11 | **-2.72** | **-4.31** | **-3.85** | **-8.52** |

^a^ Log_2_ FC relative to EtOH. Statistically significant differences are indicated in bold text (FDR < 0.05)
