# Supplementary material for: Epigenetic weapons in plant-herbivore interactions: Sulforaphane disrupts histone deacetylases, gene expression, and larval development in Spodoptera exigua while the specialist feeder Trichoplusia ni is largely resistant to these effects
Source: PLoS One. 2023 Oct 19;18(10):e0293075. doi: 10.1371/journal.pone.0293075 (PMC10586618; doi:10.1371/journal.pone.0293075)
Supplement: S1 Fig — In a common experiment we cataloged gene expression changes in both species in response to SFN, compared to ethanol alone (negative control) and the pharmaceutical HDACi TSA (positive control). Exposure to dietary SFN reduced the size of S. exigua larvae but not T. ni larvae, as observed in previous experiments. A two-factor ANOVA confirmed that larval size was impacted by the consumption of SFN and/or TSA (P<0.001) and by species (P<0.001). Letters indicate pair wise differences detected by Student-Newman-Keuls multiple comparisons tests. (DOCX) [file pone.0293075.s002.docx]

**Supplementary Information Appendix for:**

**Epigenetic weapons in plant-herbivore interactions: Sulforaphane disrupts lepidopteran histone deacetylases, gene expression, and larval development**

Dana J. Somers, David B. Kushner, Alexandria R. McKinnis, Dzejlana Mehmedovic, Rachel S. Flame, and Thomas M. Arnold*

Department of Biology, Program in Biochemistry and Molecular Biology, Dickinson College, Carlisle, PA USA 17013

*corresponding author: [arnoldt@dickinson.edu](mailto:arnoldt@dickinson.edu)

**S1 Figure. Consumption of SFN altered the expression of genes in fat body tissues of *S. exigua* and, to a lesser degree, *T. ni*.** In a common experiment we cataloged gene expression changes in both species in response to SFN, compared to ethanol alone (negative control) and the pharmaceutical HDACi TSA (positive control). Exposure to dietary SFN reduced the size of *S. exigua* larvae but not *T. ni* larvae, as observed in previous experiments. A two-factor ANOVA confirmed that larval size was impacted by the consumption of SFN and/or TSA (P<0.001) and by species (P<0.001). Letters indicate pair wise differences detected by Student-Newman-Keuls multiple comparisons tests.
