# Supplementary material for: Epigenetic weapons in plant-herbivore interactions: Sulforaphane disrupts histone deacetylases, gene expression, and larval development in Spodoptera exigua while the specialist feeder Trichoplusia ni is largely resistant to these effects
Source: PLoS One. 2023 Oct 19;18(10):e0293075. doi: 10.1371/journal.pone.0293075 (PMC10586618; doi:10.1371/journal.pone.0293075)
Supplement: S2 Fig — Color intensity reflects the degree of expression changes, highlighting the greater magnitude of expression changes in S. exigua, compared to T. ni. Hierarchical clustering based on Euclidean distance of 1,792 S. exigua (A) and 2,454 T.ni (B) genes significantly differentially expressed after exposure to either SFN or TSA. Values represent the log2 read counts per million mapped reads (CPM). Data are scaled such that each gene has mean = 0 and standard deviation = 1. (DOCX) [file pone.0293075.s003.docx]

**Supplementary Information Appendix for:**

**Epigenetic weapons in plant-herbivore interactions: Sulforaphane disrupts lepidopteran histone deacetylases, gene expression, and larval development**

Dana J. Somers, David B. Kushner, Alexandria R. McKinnis, Dzejlana Mehmedovic, Rachel S. Flame, and Thomas M. Arnold*

Department of Biology, Program in Biochemistry and Molecular Biology, Dickinson College, Carlisle, PA USA 17013

*corresponding author: [arnoldt@dickinson.edu](mailto:arnoldt@dickinson.edu)


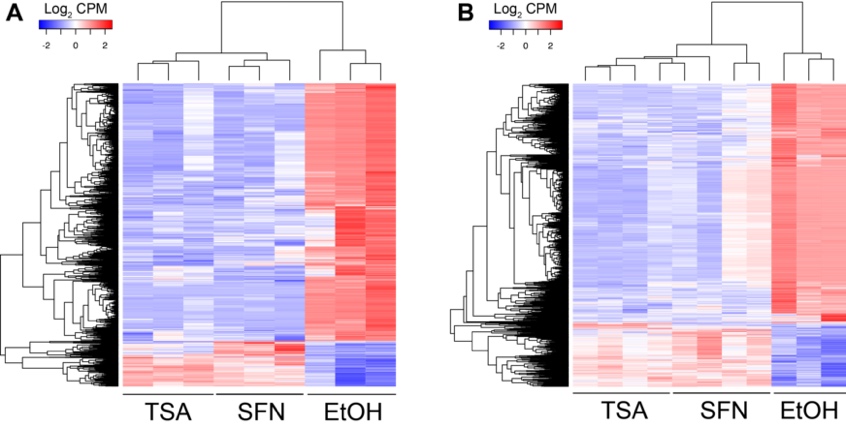


**S2 Figure. Significantly differentially expressed genes in *S. exigua* and *T. ni*.** Color intensity reflects the degree of expression changes, highlighting the greater magnitude of expression changes in *S. exigua*, compared to *T. ni*. Hierarchical clustering based on Euclidean distance of 1,792 *S. exigua* (**A**) and 2,454 *T.ni* (**B**) genes significantly differentially expressed after exposure to either SFN or TSA. Values represent the log_2_ read counts per million mapped reads (CPM). Data are scaled such that each gene has mean = 0 and standard deviation = 1.
