# Supplementary material for: Epigenetic weapons in plant-herbivore interactions: Sulforaphane disrupts histone deacetylases, gene expression, and larval development in Spodoptera exigua while the specialist feeder Trichoplusia ni is largely resistant to these effects
Source: PLoS One. 2023 Oct 19;18(10):e0293075. doi: 10.1371/journal.pone.0293075 (PMC10586618; doi:10.1371/journal.pone.0293075)
Supplement: Attachment — Submitted filename: Response to reviewers (final).pdf [file pone.0293075.s004.pdf]

Response to editor and reviewers

We are grateful to the reviewers for their comments and suggestions, which we have used to improve the manuscript.

As per the reviewer's suggestions, we have changed the title of the manuscript to be:

EPIGENETIC WEAPONS IN PLANT-HERBIVORE INTERACTIONS: SULFORAPHANE DISRUPTS ~~LEPIDOPTERAN~~ HISTONE DEACETYLASES, GENE EXPRESSION, AND LARVAL DEVELOPMENT <u>IN *SPODOPTERA EXIGUA* WHILE THE SPECIALIST FEEDER TRICHOPLUSIA NI IS LARGELY RESISTANT TO THESE EFFECTS</u>

We observed that some of the questions may have arisen from misunderstandings or a desire for more information. In an attempt to be clearer we have included additional information here.

<u>Formatting corrections</u>

- We have removed the funding information from the Acknowledgments section. It remains in the Funding Statement, as prescribed.
- ORCID iD included for the corresponding author in Editorial Manager.
- We have revised Figure 1 to remove copyrighted image and clarify the experimental design, as suggested by reviewers.

<u>Responses to Reviewer 1</u>

Reviewer 1. *"The title of the paper, "Epigenetic Weapons in Plant-Herbivore Interactions," implies that the study focuses on the effects of sulforaphane (SFN) on feeding behavior in a specialist Trichoplusia ni and a generalist Spodoptera exigua. However, the results indicate that SFN does not have any negative impact on feeding, development, or mortality in T. ni."*

<u>In an effort to be clearer we have revised the title of the manuscript.</u>

To clear up a possible misunderstanding please note that:

1. Both species were affected, one way or another. For example, there were SFN-induced gene expression changes observed in both species (see Figure 5). One species was simply impacted to a greater degree, and in some different ways, compared to the other.

2. In terms of larval development there were indeed differences; at these concentrations SFN slowed the development of the generalist, *S. exigua*, but not the specialist, *T. ni* (see Figure 2).

3. But SFN did not have a negative effect on feeding or mortality in <u>either</u> species (see Figure 2).

Reviewer 1. *"It is worth noting that a specialist pest typically causes more damage to a plant than a generalist. As such, the results presented in the paper are insufficient to justify the title. To support the hypothesis that S. exigua consumes more plant tissue than T. ni, a feeding/non-feeding choice assay can be conducted."*

Our apologies if we have not fully understood this comment. We did not hypothesize that *S. exigua* consumes more plant tissue than *T. ni*. We're not sure how this relates to the title. No plants or plant tissues were studied in these experiments.

We are not aware of evidence that specialist pests cause more damage to a plant than generalists. While this seems reasonable in some cases it would almost certainly depend on the plant, the herbivores, and the situation.

Reviewer 1. "Similarly, the title "Sulforaphane Disrupts Lepidopteran Histone Deacetylases" is somewhat misleading, as the activities of HDAC enzymes were not inhibited, and development was not disrupted in the lepidopteran insect T. ni. Given that generalists are typically more sensitive to plant toxins than specialists, this finding is noteworthy."

As described above, we have changed the title.

To help explain the reason for the original title we note that <u>HDAC enzymes from both species were equally inhibited</u> by SFN *in vitro (*see Figure 3). The differences were observed in the *in vivo* experiments with living larvae; here we observed that the specialist herbivore probably benefited from a mechanism for detoxifying SFN before it reaches HDAC enzymes.

Reviewer 1. It should be noted that the family Cruciferae is now known as Brassicacea and should be updated in the manuscript.

We think there was a misunderstanding. The word Cruciferae did not appear in our manuscript. We never mentioned the family of the plants. We described these plants in the introduction thusly:

SFN is produced by plants in the order Brassicales (the cruciferous or mustard oil plants) including cabbages, broccoli, Brussels sprouts, bok choy, cauliflower, rapeseed, watercress, and Arabidopsis [6,62,73–76]. This group includes the most important cultivated leafy vegetables [67].

Reviewer 1. The abstract states that cruciferous plants produce sulforaphane (SFN), an inhibitor of nuclear histone deacetylases (HDACs). However, it is important to clarify that SFN is not produced constitutively by Brassicacea plants. It is a product of the Mustard Oil Bomb system of Brassicacea, which is activated by herbivore or mechanical damage.

We did not state that SFN is produced constitutively, in the abstract or elsewhere in the manuscript.

On the contrary, we indicate that SFN is produced as a product of the "mustard oil bomb" only when these plants are chewed or crushed. We described the mustard oil bomb reactions in lines 160-168 of the original manuscript:

"When these plants are chewed or crushed, a "mustard oil bomb" reaction is generated in which glucosinolates are hydrolyzed by plant myrosinases (ß-thioglucoside glucohydrolases) to generate nitriles, isothiocyanates, thiocyanates, oxazolidine-2-thiones, and epithionitriles [6,73,74,77,78]. These products can serve as defenses against herbivores [67,79–88] and influence the taste and flavor of cruciferous plants. SFN is the hydrolysis product of one particular glucosinolate, glucoraphanin (4-(methylsulfinyl) butyl glucosinolate) [61,62,64]. Most insect herbivores feeding on cruciferous plants would be exposed to SFN with every bite, though some possess adaptations to deactivate or redirect the mustard oil bomb reactions [80,88–90]."

Reviewer 1. The introduction of the manuscript discusses epigenetic regulation and HDAC inhibitors but fails to fully elucidate the role of SFN in herbivory and the reasoning behind its selection.

The role of SFN in herbivory has not been well studied, probably because it is a generally a minor product of the mustard oil bomb reactions. It has been largely ignored. Its potential role as an inhibitor of epigenetic systems has not been explored. As per line 169-170: *"…as far as we are aware, the impacts of SFN on the epigenetic systems of insect herbivores have not been investigated."*

The fact that the effects of SFN on the epigenetic systems of herbivores has not been investigated is quite surprising. SFN has long been known to be an effective HDAC inhibitor in humans, altering the expression of hundreds of genes, and we wondered what it was doing to herbivores in nature, which may consume SFN with every bite.

Here we show that SFN does indeed work this way in (at least some) herbivorous insects. Our conclusion is that investigators should examine the role of SFN in herbivory which may be larger – and more interesting - than we thought.

<u>We note that Reviewer 2 offered the opposite advice, that we should remove the "reasoning" from the introduction.</u>

Reviewer 1. The illustration in Figure 1, which depicts the basic design of single generation experiments, is somewhat unclear. It could be made more informative and illustrative.

Thank you for this suggestion. In the revised manuscript, <u>we have made revisions to Figure 1 to make the experimental design clearer</u>. The specifics of individual experiments differ a bit, of course. Additional details are described in the methods section.

Reviewer 1. To confirm whether SFN affects HDAC, quantitative real-time analysis of HDAC-related genes should be conducted, using appropriate housekeeping genes prior to enzyme activity and RNA seq. This will help to validate the upregulation or downregulation of HDAC genes.

In the past, it was standard practice to use qRT-PCR analyses to confirm RNAseq results. However, modern RNAseq analyses are quite powerful and nearly as sensitive as qRT-PCR. Recent studies indicate that the two technologies provide similar results in 98% of cases, with less than 2% of genes yielding "severely non-concordant" results (e.g., Everaert et al 2017 *"Benchmarking of RNA-sequencing analysis workflows using whole-transcriptome RT-qPCR expression data"*). In the <2% of cases where there was a disagreement between the methods this tended occur for genes with very low expression levels. Another comparative study concluded that "If all experimental steps and data analyses are carried out according to the state-of-the-art, results from RNA-seq are expected to be reliable and if they are based

on a sufficient number of biological replicates, the added value of validating them with qPCR (or any other approach) is likely to be low" (Coenye T. 2021 "*Do results obtained with RNA-sequencing require independent verification?*"). In this case, our results were the same across several different experiments and RNAseq analyses, each with adequate biological replication. The gene expression levels were not unusually low. Thus, it seems unnecessary to use qRT-PCR analyses to confirm these RNAseq results.

Reviewer 1. SFN feeding slowed the development of S. exigua. This slow larval development can contribute to increased feeding time by the herbivore when feeding on Brassicacea plants. However, pupation and mortality of S. exigua were unaffected by SFN treatments. As such, the results presented in the manuscript are insufficient to justify the title. The title could be revised to reflect the differences in feeding behavior between a generalist and a specialist.

We have changed the manuscript title and some of the subheadings, as suggested.

To clear up any misunderstanding, there were <u>no observed differences in the feeding behavior between the generalist and the specialist</u>, with or without the SFN or TSA treatments (see Methods section). Also, there were no effects on pupation or mortality. That is not how SFN seems to work. Here we are only stating that SFN acts to inhibit HDAC enzymes of some herbivores (see Figure 3), inducing large scale changes in gene expression (see Figure 5).

8. It is unclear what the rationale behind SFN application to egg masses is and how this correlates with natural herbivore feeding.

Thank you, we should have explained this more fully in the methods.

We considered the possibility that SFN was distasteful, and might alter feeding rates, causing larvae to eat less and, therefore, grow slowly. We did not observe this to be the case (as mentioned in the method section). Nonetheless it seemed prudent to find another way to treat the organisms, one which eliminated this potential confounding factor. At first, we attempted to treat tiny first-instar larvae with a droplet of SFN in a penetrating solvent, such as DMSO. However, at this stage the larvae are so small that they drown in the droplet. Applying SFN topically to older larvae doesn't allow enough time for the response to emerge. So, we opted to treat eggs. In our experience, the eggs soak up SFN well and hatch normally. They respond to this treatment just as they do to consumption of SFN in their diets.

In the original manuscript we mention this briefly in the discussion:

The method of SFN treatment did not seem to matter: similar responses were observed when SFN was consumed in the diet and when eggs were treated topically with SFN, supporting the hypothesis that SFN acts directly as a HDAC inhibitor rather than affecting feeding rates.

However, we agree that this should be expanded, and mentioned earlier. <u>We have added text to the methods section to further explain the rationale:</u>

We considered the possibility that SFN and TSA could be distasteful, suppressing feeding and, therefore growth rates. We did not observe this to be the case in any experiments. Nonetheless, it seemed prudent to run parallel experiments wherein these substances were applied topically, rather than via the

diet. As a result, we also conducted similar experiments wherein HDAC inhibitors were applied topically to eggs, using DMSO as a penetrating solvent.

Reviewer 1. The SFN treated and untreated larval development should be compared using a paired t-test that student's t-test.

These samples were not paired. In most experiments, there were three or more groups (control, SFN-treated, and TSA-treated). Hence, paired sample t-tests are not appropriate, or possible.

Reviewer 1. Interestingly, the emergence of T. ni moths was not delayed but rather was accelerated by approximately two days in both TSA and the highest concentrations of SFN (Figure 2D). Despite these effects on larval development, there were no detected differences in pupal mass or the size, appearance, and behavior of adult moths in any treatment group. The authors have not provided an explanation for this observation.

This is correct. We observed in this experiment that *T. ni* larval development time was accelerated by about two days yet, ultimately, the pupal masses and the appearance and behavior of moths were not different. It just took a shorter amount of time to reach pupation.

We made similar observations when larval development was slowed in *S. exigua*. It just took longer to reach pupation.

In each case, the larvae seem to pupate whenever they are large enough, on their own schedule, and this didn't seem to affect the later stages.

We agree that this is interesting. However, our experiments were not designed to provide an explanation. Here we simply observed that the length of the larvae stage was indeed extended. This can affect predation and thus mortality (*a la* the slow growth – high mortality hypothesis), so there may be real consequences for survival in nature.

Reviewer 1. In Figure 3B, nuclear and cytoplasmic HDAC enzymes were extracted from S. exigua (red) and T. ni (blue), and their activities were quantified in vitro in the presence of various concentrations of SFN. The artificial diet containing the dissolved ethanol concentration is not shown, but its inclusion will help to elucidate the exact effect of SFN on nuclear and cytoplasmic HDAC enzymes.

To clear up this misunderstanding, we would call the reviewer's attention to figure 3B. The HDAC enzyme activity is plotted over a range of SFN concentration and the zero concentration is the ethanol control group. They are the left-most data points. These are log scale axes, so the X-axis shows 0.01 M not 0 M at the low end. (The logarithm of zero is not defined; hence, it is mathematically impossible to plot zero on a log scale.) To clarify this, we have added the following text to the Figure 3 caption: "Con. indicates use of the EtOH carrier solvent in (A) while the leftmost data points represent the control in (B)."

Reviewer 1. Table 1 can be presented more informatively in the form of graphs rather than a table.

We have created such a figure and found that it conveys less information compared to the table. Important information is lost. Specifically, presenting these data in the form of a table allows readers to

more precisely compare the numerical differences of four different categories (P value, number of genes in the cluster, number of genes in the genome, and fold enrichment over background). If a graph were used, the data would have to be presented on multiple Y-axes, or in multiple graphs, (because the scale of these numbers are so different), and thus it would be challenging to compare across these categories for each function. Furthermore, the P-values range from 6.41E-35 to 0.045, and any graph would lose the precision of these numbers.

Reviewer 1. Third, we considered the possibility that the resistance of T. ni in our experiments compared to S. exigua was due to differences in the insects' HDAC enzymes themselves; however, this does not seem to be the case.

This is a section of text copied from the manuscript (second paragraph of section 2 in the Discussion). But no question followed. Was there additional text that was cut off?

Responses to Reviewer 2

For the INTRODUCTION, I would suggest to provide necessary background first and propose the hypothesis and reasoning at the end. Too much reasoning was mixed with the background information, which might influence the flow of the INTRODUCTION. In addition, I do not think DNA methylation and non-coding RNA is necessary to appear. However, the detailed information for the histone modifications would be required.

Thank you. This is something we struggled with.

In the introduction we aimed to connect two fields that are usually quite distinct: plant chemical defense and epigenetics (or, more generally, ecology and medicinal science). This is why we chose to submit to PLoS ONE rather than a disciplinary journal; we felt we needed the flexibility to explain this interdisciplinary work.

The reasoning, or rationale for doing this, connects these two different sections of the introduction. We have tried other approaches, *e.g.*, changing the order in which the topics are presented, but found it to be a circular puzzle. In the end, one topic has to come before the other, and the reasoning links them together. Without the reasoning it falls apart.

We also saw that reviewer 1 requested more reasoning/rationale in the introduction.

Regarding the mentions of DNA methylation and non-coding RNA, this is a good point. We didn't explore these other two mechanisms. We mentioned them for a couple of reasons: (1) the field of insect epigenetics is finally growing rapidly and is exploring all three types of epigenetic systems and (2) having established (we hope) that some plant products act to disrupt one of these systems we are hoping readers will see how broadly this could be occurring in nature, as all three epigenetic systems would be vulnerable to sabotage by clever plants. We wanted to make the term "epigenetic weapon" a broad enough category that in the future in might encompass substances that interfere with histone acetylation, DNA methylation, or the action of non-coding RNAs. We hope it is acceptable to mention all three of these systems in the introduction. Two of the systems – DNA methylation and non-coding RNAs - are mentioned only briefly. A couple of sentences each.

Reviewer 2. The authors just used the fat body to quantify HDAC enzyme activities or perform RNA-seq. Because midgut is also an important tissue in plant-insect interaction, I would suggest to provide some reasoning and discussion for such practice.

Thank you. Yes, midgut tissue is sometimes used for this purpose. We did not use it here for a couple of reasons:

(1) We can't. Midgut tissue would contain some artificial diet and, thus, some SFN or TSA. These are, of course, HDAC enzyme inhibitors making it impossible for use to accurately measure the activity of these enzymes in these tissues, which was a goal. (In fact, TSA is used in the enzyme assays to create a blank, with zero enzyme activity. It is quite powerful and even traces would interfere.) We could try to collect and clean midgut tissue but we'd never be certain we eliminated all of the SFN or TSA in the samples.
(2) It is not necessary. Studies of other plant natural products study midgut tissues because they are guaranteed to come in contact with substances present in the diet, even if they are not well absorbed. However, SFN is well absorbed and becomes distributed throughout animal tissues. It even passes the blood-brain barrier.
(3) Fat body tissue is important in larval development which we aimed to study; it expands as fat stores increase and later helps to power metamorphosis.
(4) Fat body tissue is relatively easy to obtain, even from larvae that are small. And larvae treated with SFN and TSA can be very small! When we use higher concentrations or more powerful HDAC inhibitors we can essentially keep some larvae at the first instar stage indefinitely. Those are quite difficult to dissect. It is ironic that the better the treatments work, the smaller some of the larvae are, and the harder it is for us to collect the tissue.
(5) We sometimes also examine ovaries; however, this requires a doubling (or more) of sample sizes since, of course, they occur only in about half of the larvae. You can never really guarantee there will be enough ovaries in a population.

<u>In order to provide more clarity as to why we used fat body tissue and not midgut tissue we added the following text under the subheading "Sample collection" in the methods section:</u>

We focused on these tissues because they are important in development and reproduction, because they can be obtained cleanly from larvae that remain smaller than usual, and because SFN is expected to reach them relatively quickly. We did not examine midgut tissue, commonly examined in similar studies, because gut tissue would likely be contaminated with HDAC inhibitors from the artificial diet, making accurate measurements of HDAC enzyme activity problematic.

Reviewer 2. The authors claimed that the magnitude of the response to the HDAC inhibitors was greater in Spodoptera exigua compared to Trichoplusia ni (Figure 5A). Can the authors provide some more details on how to get this conclusion?

Thank you, this is an important point to emphasize. The number of genes affected in *S. exigua* and *T. ni* were roughly similar. But <u>the degree</u> to which their expression was altered was dramatically different. <u>The gene expression of *S. exigua* was altered to a much greater extent.</u>

Hence, the statement from the original manuscript, lines 366-368:

Importantly, although the response patterns were similar for both species, ***the magnitude of the response to these HDAC inhibitors was greater in S. exigua compared to T. ni*** *(Figure 5A).*

And statements in lines 459-465 in the Discussion of the original manuscript:

The consumption of SFN affected the expression of thousands of genes. Overall, the patterns of differentially expressed genes were similar in both species in response to SFN and TSA, suggesting that SFN does indeed act primarily as an HDAC inhibitor when consumed in the diet at natural concentrations. ***Although the patterns of gene expression were similar, the magnitude of changes was generally greater in S. exigua as compared to T. ni. (Figure 5). In short, similar genes were affected but to a lesser degree in T.ni, corresponding with the resistance of this species that we observed in these experiments.***

From the abstract:

RNA-seq analyses revealed that the consumption of SFN alters gene expression in T. ni in similar ways, but to a lesser degree, compared to S. exigua.

This can also be seen visually in the heat maps in the supplementary materials. We have added the following sentences to that caption on the heat maps presented in the supplementary material to help made this clearer:

S1 Figure. Significantly differentially expressed genes in *S. exigua* and *T. ni*. Color intensity reflects the degree of expression changes, highlighting the greater magnitude of expression changes in *S. exigua*, compared to *T. ni.* Hierarchical clustering based on Euclidean distance of 1,792 S. exigua (A) and 2,454 T.ni (B) genes significantly differentially expressed after exposure to either SFN or TSA. Values represent the log2 read counts per million mapped reads (CPM). Data are scaled such that each gene has mean = 0 and standard deviation = 1.

(question continued) In addition, the authors identified 181 and 409 genes in S. exigua and T. ni, respectively. What did this mean? Since SFN inhibited the HDAC enzymes in S. exigua but not T. ni, how could this type of comparison shown in Venn diagram make sense?

For the most part, SFN and TSA elicited changes in the same collection of genes in both species (Figures 5B,C). However, there were a small number of species-specific differences. For example, there were 181 genes repressed by SFN only in *S. exigua*. And there were 409 genes repressed by SFN only in *T. ni*. Therefore, we noted that "we also identified 181 and 409 genes in *S. exigua* and *T. ni*, respectively that showed species-specific repression". The main point is that the majority of differential expressed genes occurred in both species, with only minor differences.

So SFN impacted the same collection of genes, for the most part, in both species. The key difference was the magnitude of the gene expression changes, which were much larger in *S. exigua* compared to *T. ni.*

Reviewer 2. Minor issues:

L136, please specify which species was studied for the 18 recognized HDAC enzymes. The list of 18 recognized HDAC enzymes come from a variety of studies focusing on a range of eukaryotes. The references cited list the species used by the various studies.

L220, please clarify in what cases ovaries were also collected? They were collected for most of these experiments. We present some of the ovary data here. Some will be presented in a forthcoming paper. The ovary data presented here comes from experiments described in Figure 2.

L320, $\alpha=0.05$? The cut off for determined significant differences was set at the standard $\alpha=0.05$ in all experiments.

L341, the appearance of section “effects of SFN on both species under different temperature” should be earlier We tried this; however, it seems to create more confusion, interrupting the flow of other sections. The current framework discusses all experiments run at the standard temperature together, then introduces temperature as another factor to consider in this last section.
